# Luteolin 7-Sulfate Attenuates Melanin Synthesis through Inhibition of CREB- and MITF-Mediated Tyrosinase Expression

**DOI:** 10.3390/antiox8040087

**Published:** 2019-04-04

**Authors:** Seok Won Lee, Jae Heon Kim, Hyerim Song, Jin Kyung Seok, Seong Su Hong, Yong Chool Boo

**Affiliations:** 1 Department of Molecular Medicine, Cell and Matrix Research Institute, BK21 Plus KNU Biomedical Convergence Program, School of Medicine, Kyungpook National University, 680, Gukchaebosang-ro, Jung-gu, Daegu 41944, Korea; seokwon2891@daum.net (S.W.L.); rkrsh@naver.com (J.H.K.); happyhyerim@knu.ac.kr (H.S.); haute87@naver.com (J.K.S.); 2 Bio-Center, Gyeonggido Business & Science Accelerator (GBSA), Suwon 16229, Korea; bestgene@gbsa.or.kr

**Keywords:** melanin, luteolin 7-sulfate, luteolin, tyrosinase, flavonoid

## Abstract

Antioxidants with antimelanogenic activity are potentially useful for the attenuation of skin hyperpigmentation disorders. In a previous study, luteolin 7-sulfate isolated from *Phyllospadix iwatensis* Makino, a marine plant, was shown to inhibit cellular melanin synthesis. The aim of the present study was to examine its action mechanism, focusing on the regulation of tyrosinase (TYR) expression in cells. Cell-based assay was undertaken using murine melanoma B16-F10 cells and primary human epidermal melanocytes (HEMs). Luteolin 7-sulfate showed lower toxicity compared to luteolin in B16-F10 cells. At the non-toxic concentration ranges, luteolin 7-sulfate attenuated melanin synthesis, stimulated by α-melanocyte-stimulating hormone or forskolin. Luteolin 7-sulfate attenuated forskolin-induced microphthalmia-associated transcription factor (MITF) and TYR expressions at the mRNA and protein levels in B16-F10 cells. It also attenuated the phosphorylation of cAMP-responsive element binding protein (CREB) stimulated by forskolin. Luteolin 7-sulfate also attenuated melanin synthesis in primary HEMs. This study demonstrates that luteolin 7-sulfate attenuates TYR gene expression through the intervention of a CREB- and MITF-mediated signaling pathway, leading to the decreased melanin synthesis.

## 1. Introduction

As a dark pigment present in skin, hair, eyes, and other tissues, melanin contributes not only to human appearance but also to skin homeostasis [1]. A variety of factors like hormonal changes and nutritional status affect skin melanin synthesis, and hypo- or hyper-pigmentation can be caused by the disruptions in melanogenesis [2]. Melasma, freckles, and senile lentigines result from an uneven distribution or abnormal accumulation of melanin in the skin, and such pigmentation patterns are sometimes undesired by many people pursuing aesthetic ideals. Thus, controlling skin hyperpigmentation is an important issue in dermatology and cosmetics. 

Melanin synthesis is directed by microphthalmia-associated transcription factor (MITF) [3]. MITF is activated in response to external stimuli by multiple mechanisms, including cAMP-responsive element binding protein (CREB), Wnt, glycogen synthase kinase 3β, and mitogen activated protein kinases, and in turn modulates the expression of melanogenic enzymes such as tyrosinase (TYR). TYR catalyzes the oxidation of l-tyrosine or l-3,4-dihydroxyphenylalanine (l-DOPA) to DOPA-quinone, the rate limiting step of melanin synthesis. Cellular melanin synthesis can be attenuated by inhibition of TYR catalytic activity and/or by suppression of TYR expression. 

Various natural and semi-synthetic compounds have been reported to inhibit cellular melanin synthesis. In our previous studies, p-coumaric acid was found to be a potent and selective inhibitor of human TYR [4,5,6], showing antimelanogenic effects in cells [7] and a depigmenting effect in human skin [8]. Resveratrol has been shown to attenuate cellular melanin synthesis via a variety of mechanisms, including the regulation of TYR protein expression and maturation, and the direct inhibition of TYR catalytic activity [9,10,11]. In addition, its semi-synthetic derivatives, such as resveratryl triacetate and resveratryl triglycholate, showed antimelanogenic effects in cells [9,12] and depigmenting effects in human skin [13,14,15]. 

Recent studies in other laboratories suggest that plant compounds are potentially useful in controlling the production of melanin in animal cells. Vitexin-2’’-O-rhamnoside extracted from the leaves of *Crataegus azarolus* L. inhibited the growth of B16F10 melanoma cells and decreased the melanin content by inhibiting TYR activity [16]. The hydroalcoholic extract of *Spartium junceum* L. flowers inhibited melanogenesis in B16-F10 cells by reducing the gene expression of melanogenesis-related genes such as MITF and TYR [17]. Tricin isolated from young green barley (*Hordeum vulgare* L.) inhibited melanin synthesis in in B16 melanoma cells more strongly than other similar compounds, such as tricetin, tricetin trimethyl ether, luteolin, and apigenin [18]. 

In the previous study [19], the extract of *Phyllospadix iwatensis* Makino was found to inhibit TYR catalytic activity the most out of the 50 different marine plant extracts tested. In addition, the active compound of *Phyllospadix iwatensis* that inhibited TYR catalytic activity was identified as luteolin 7-sulfate. The purpose of the present study was to further examine its antimelanogenic effects, focusing on TYR expression. For this purpose, we synthesized luteolin 7-sulfate from luteolin, and examined its effects on melanin contents, the mRNA and protein expressions of TYR and MITF, and the phosphorylation of CREB in cultured melanocytic cells.

## 2. Materials and Methods 

### 2.1. Reagents

Luteolin (purity > 98%), N,N’-dicyclohexyl carbodiimide, tetrabutyl ammonium hydrogen sulfate, α-melanocyte-stimulating hormone (α-MSH), forskolin, dimethyl sulfoxide (DMSO), and DMSO-d_6_ were purchased from Sigma-Aldrich (St. Louis, MO, USA).

### 2.2. Instrumental Analysis

High performance liquid chromatography (HPLC) was performed using a Gilson HPLC system (Gilson, Inc., Middleton, WI, USA) with a 321 pump and a UV/VIS 151 detector. Separation was carried out on a 5 μm Hector-M C_18_ column (4.6 mm × 250 mm) (RS tech co., Daejeon, Korea), using a mobile phase consisting of 0.5% formic acid (A) and acetonitrile (B). The gradient program was set up as follows: 0–30 min, linear gradation of 20–80% B; 30–35 min, 80–100% B; 35–40 min, 100–20% B; 40–50 min, 20% B. The flow rate was 0.6 mL min^−1^. The detector was set at 350 nm. The ultraviolet (UV) absorption spectrum was measured with a Shimadzu UV-1650PC spectrophotometer (Shimadzu Corporation, Kyoto, Japan). Nuclear magnetic resonance (NMR) spectra were measured on a Bruker Ascend III 700 spectrometer (Bruker BioSpin, Rheinstetten, Germany). Tetramethylsilane (TMS) was used as the internal standard, and chemical shifts were indicated by δ values. Electrospray ionization mass spectra (ESI-MS) were obtained using an Agilent 6130 Quadrupole liquid chromatography/mass spectrometer (Agilent, Santa Clara, CA, USA).

### 2.3. Synthesis and Purification of Luteolin 7-Sulfate

Luteolin 7-sulfate was chemically synthesized from luteolin, as previously described [20]. The reaction mixture contained 10 eq. N, N’-dicyclohexyl carbodiimide, 1 eq. luteolin, and 2 eq. tetrabutyl ammonium hydrogen sulfate in pyridine (5.6 mL), and reacted for 16 h at 4 °C. The resulting reaction mixture was diluted two-fold with methanol and centrifuged to remove the precipitate. The supernatant was loaded on a Sephadex LH20 (Sigma-Aldrich) column (3 cm × 40 cm) and eluted with methanol. The fractions containing the desired product were collected and loaded on a column (3 cm × 10 cm) of cation-exchange resin (Dowex 50WX8, H^+^ form, Sigma-Aldrich), preconditioned with 1 M K_2_CO_3_ (200 mL) and eluted with water. The eluate was concentrated under reduced pressure to dryness. Luteolin 7-sulfate: yellow crystalline powder: UV (EtOH) λ_max_ (log ε), 253 nm (3.95), 348 nm (4.00); ^1^H-NMR (700 MHz, DMSO-d_6_) and ^13^C-NMR (175 MHz, DMSO-d_6_) data, see Table 1; ESI-MS (negative mode) *m/z* 365 [M]^−^. 

### 2.4. Cell Culture

Murine melanoma B16-F10 cells were purchased from the American Type Culture Collection (ATCC) (Manassas, VA, USA) and human epidermal melanocytes (HEMs) derived from moderately pigmented neonatal human foreskins were purchased from Cascade Biologics (Portland, OR, USA). These cells were cultured as previously described [19]. Cells were seeded into six-well culture plates at a density of 1.2 × 10^5^ cells per well and incubated for 24 h. Cells were then treated with various concentrations of the test substance and stimulated with 0.1 μM α-MSH or 10 μM forskolin for the specified time. Cell viability was measured using the 3-(4,5-dimethylthiazol-2-yl)-2,5-diphenyl tetrazolium bromide (MTT) assay.

### 2.5. Melanin Content Assay

The amount of melanin retained in the cells (intracellular melanin) and that was secreted into cultured medium (extracellular melanin) was determined by a spectrophotometric method [21]. The melanin content was normalized to the total protein content of the cells, which was determined using the Bio-Rad DC assay.

### 2.6. TYR Activity Assay

Cells were suspended in an ice-cold lysis buffer containing 10 mM Tris-HCl (pH 7.4), 120 mM sodium chloride, 25 mM potassium chloride, 2.0 mM ethylene glycol tetraacetic acid, 1.0 mM ethylene diamine tetraacetic acid, 0.5% Triton X-100, and a protease inhibitor cocktail (Roche, Mannheim, Germany), and centrifuged at 13,000 × g for 15 min at 4 °C to obtain cell-free extracts. TYR activity was determined using l-tyrosine plus l-DOPA [19,22]. The reaction mixture (200 μL) consisted of 100 mM sodium phosphate (pH 6.8), 1.0 mM l-tyrosine, 42 μΜ l-DOPA, and cell-free extracts (40 μg protein), and incubated at 37 °C. The changes in absorbance at 475 nm were measured using a SPECTROstar Nano microplate reader and corrected for the value without l-DOPA.

### 2.7. Western Blotting

Western blotting of whole cell lysates was performed as previously described [23]. The primary antibodies against TYR, MITF, and β-actin were purchased from Santa Cruz Biotechnology (Santa Cruz, CA, USA). Primary antibodies for CREB and phospho-CREB (Ser^133^) were from Cell Signaling Technology (Danvers, MA, USA). Secondary antibodies were from Cell Signaling (Danvers, MA, USA). Reactive bands were detected using a picoEPD Western Reagent kit (ELPIS-Biotech, Daejeon, Korea). 

### 2.8. Quantitative Reverse Transcription Polymerase Chain Reaction (qRT-PCR) Analysis

Cell RNA was isolated from B16-F10 cells using an RNeasy kit (Qiagen, Valencia, CA, USA). The complementary DNA (cDNA) was synthesized from 1 μg of RNA by reverse transcription using a High-Capacity cDNA Archive Kit (Applied Biosystems, Foster City, CA, USA). Gene-specific primers for PCR were purchased from Macrogen (Seoul, Korea). The sequences of the primers used in this study were: *TYR* (GenBank accession number NM_011661.5) 5′-CTTCTTCTCCTGGCAGAT C-3′ (forward) and 5′-TGGGGGTTTTGGCTTTGTC-3′ (reverse) [24]; *MITF* (NM_008601.3) 5′-GCTG GAAATGCTAGAATACAG-3′ (forward) and 5′-TTCCAGGCTGATGTCATC-3′ (reverse) [24]; and glyceraldehyde 3-phosphate dehydrogenase (*GAPDH*, NM_001289726.1) 5′-GCATCTCCCTCACAA TTTCCA-3′ (forward) and 5′-GTGCAGCGAACTTTATTGATGG-3′ (reverse) [25]. The qRT-PCR was conducted using a StepOnePlus™ Real-Time PCR System (Applied Biosystems). The reaction mixture (20 μL) comprised SYBR^®^ Green PCR Master Mix (Applied Biosystems), cDNA (60 ng), and the gene-specific primer sets (2 pmol). The reaction was performed using the following protocol: initial incubation at 50 °C for 2 min; DNA polymerase activation at 95 °C for 15 min; and annealing and extension at 60 °C for 1 min. In each qRT-PCR analysis, the melting curve showed a single peak, confirming homogeneity of the PCR products. The comparative Ct method [26] was used to assess the expression levels of mRNA corresponding to *TYR* and *MITF* in comparison with the level of the internal reference *GAPDH* mRNA. Ct is defined as the number of cycles required for the PCR signal to exceed the threshold level. Fold changes in the test group compared to the control group were calculated as 2^−ΔΔCt^, where ΔΔCt = ΔCt_(test)_ − ΔCt_(control)_ = [Ct_(gene, test)_ − Ct_(reference, test)_] − [Ct_(gene, control)_ − Ct_(reference, control)_].

### 2.9. Statistical Analysis

Data are presented as the mean ± SE of three independent experiments. The experimental results were statistically analyzed in SigmaStat v.3.11 statistical analysis software (Systat Software Inc, San Jose, CA, USA) by one-way analysis of variance (ANOVA), and *p* < 0.05 was considered to be statistically significant. 

## 3. Results

### 3.1. Luteolin 7-Sulfate 

Luteolin 7-sulfate was synthesized from luteolin by the method of Barron et al. [20]. Its ^1^H- NMR spectrum showed six proton signals in the aromatic region; a pair of meta coupled protons at δ_H_ 6.52 (1H, d, J = 2.1 Hz, H-6) and 7.03 (1H, d, J = 2.1 Hz, H-8), a one proton singlet at δH 6.76 (1H, s, H-3), and the AMX spin system at δ_H_ 7.47 (1H, d, J = 2.1 Hz, H-2’), 7.46 (1H, d, J = 8.4, 2.1 Hz, H-6’), and 6.89 (1H, d, J = 8.4 Hz, H-5’), which were in accordance with a luteolin derivative [27]. The ^13^C NMR and distortionless enhancement by polarization transfer (DEPT) spectra revealed 15 carbon signals, including nine quaternary carbons (one carbonyl) and six methines (Table 1). When compared to the carbon and proton values in luteolin, the same pattern was also seen in luteolin 7-sulfate and the downfield carbon data for C-6 and C-8, as well as the significantly downfield chemical shifts of H-6 and H-8 strongly indicating the presence of an electron withdrawing sulfate group at position C-7. The chemical identity of luteolin 7-sulfate was further verified by comparing it with data from the literature [20,28].

### 3.2. Effects of Luteolin, Luteolin 7-Sulfate, and Arbutin on the Viability and the Melanin Contents of B16-F10 Cells

**Figure 1:**
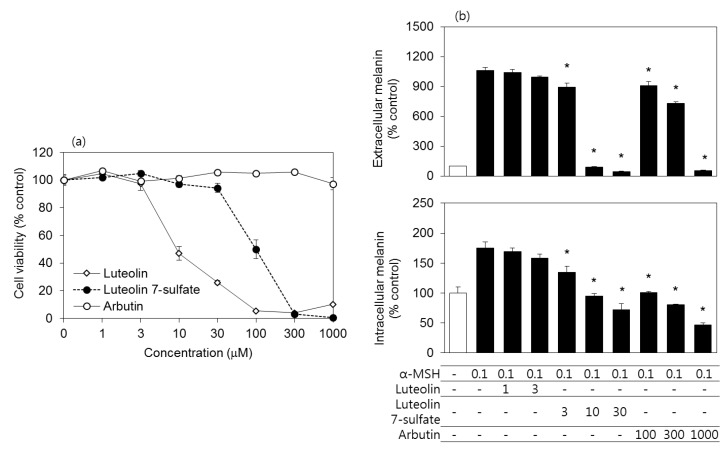
Effects of luteolin, luteolin 7-sulfate, and arbutin on the viability and melanin contents of B16-F10 cells. (**a**) Cells were treated with luteolin, luteolin 7-sulfate, or arbutin at varied concentrations, and the cell viability was determined after 48 h. (**b**) Cells were treated with luteolin, luteolin 7-sulfate, or arbutin at their non-toxic concentration ranges for 60 min and stimulated with 0.1 μM α-melanocyte-stimulating hormone (α-MSH), followed by 48 h-incubation. Extra- and intracellular melanin contents were corrected for total protein content of the cells. The means ± SEs of three independent experiments are presented. * *p* < 0.05 vs. α-MSH alone.

The cytotoxicity of luteolin 7-sulfate was compared to those of luteolin and arbutin in murine melanoma B16-F10 cells. Arbutin is known as a melanogenesis inhibitor [29], and is used as a reference in this study. As shown in Figure 1A, the cytotoxicity of luteolin 7-sulfate was much weaker than luteolin. Arbutin was non-toxic at the tested concentration ranges. Because luteolin, luteolin 7-sulfate, and arbutin showed different cytotoxicity, their antimelanogenic effects were examined at their non-toxic concentration ranges. As shown in Figure 1B, α-MSH treatment increased both the extracellular and intracellular melanin contents of B16-F10 cells, and these increases were reduced by luteolin 7-sulfate (3–30 μM) and arbutin (100–1000 μM). The antimelanogenic effect of luteolin 7-sulfate was about 10 times more potent than arbutin. 

### 3.3. Effects of Luteolin 7-Sulfate on Cellular TYR Activities and TYR Protein Levels

**Figure 2:**
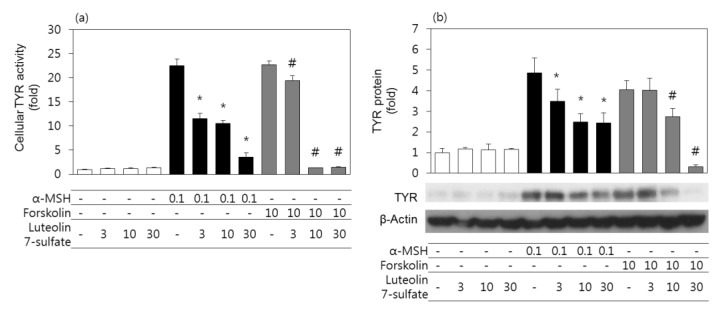
Effects of luteolin 7-sulfate on cellular tyrosinase (TYR) activities and TYR protein levels. B16-F10 cells were pretreated with luteolin 7-sulfate at varied concentrations and stimulated with 0.1 μM α-melanocyte-stimulating hormone (α-MSH) or 10 μM forskolin for 24 h. Cell lysates were used for the TYR activity assay (**a**), and western blotting of TYR protein comparing with β-actin (**b**). The means ± SEs of three independent experiments are presented. * *p* < 0.05 vs. α-MSH alone, # *p* < 0.05 vs. forskolin alone.

Experiments were then undertaken to examine the effects of luteolin 7-sulfate on the cellular TYR activities and TYR protein levels in B16-F10 cells. In addition to α-MSH, forskolin was used as an adenylate cyclase activator to stimulate cells. As shown in Figure 2A, both α-MSH and forskolin increased cellular TYR activity, and these changes were attenuated by luteolin 7-sulfate in dose-dependent manners. As also shown in Figure 2B, both α-MSH and forskolin increased TYR protein levels, and these changes were attenuated by luteolin 7-sulfate in a dose-dependent manner.

### 3.4. Effects of Luteolin 7-Sulfate on TYR and MITF mRNA Levels

**Figure 3:**
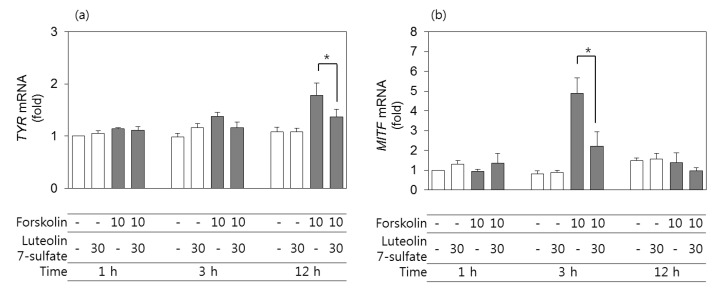
Effects of luteolin 7-sulfate on the mRNA levels of tyrosinase (*TYR*) and microphthalmia-associated transcription factor (*MITF*). B16-F10 cells were pretreated with 30 μM luteolin 7-sulfate and stimulated with 10 μM forskolin for 1, 3, and 12 h. The mRNA levels of *MITF* (**a**) and *TYR* (**b**) were determined by quantitative reverse transcription polymerase chain reaction and normalized with respect to glyceraldehyde 3-phosphate dehydrogenase. The means ± SEs of three independent experiments are presented. * *p* < 0.05.

The expression levels of the mRNAs corresponding to *TYR* and *MITF* in B16-F10 cells were assessed by qRT-PCR using *GAPDH* as a control. As shown in Figure 3A and B, the expression level of *TYR* mRNA increased after 12 h of treatment with forskolin, and the expression level of *MITF* mRNA increased after 3 h of treatment with forskolin. More importantly, these changes were attenuated in cells pretreated with 30 μM luteolin 7-sulfate. 

### 3.5. Effects of Luteolin 7-Sulfate on MITF Protein and Phospho-CREB Protein Levels

**Figure 4:**
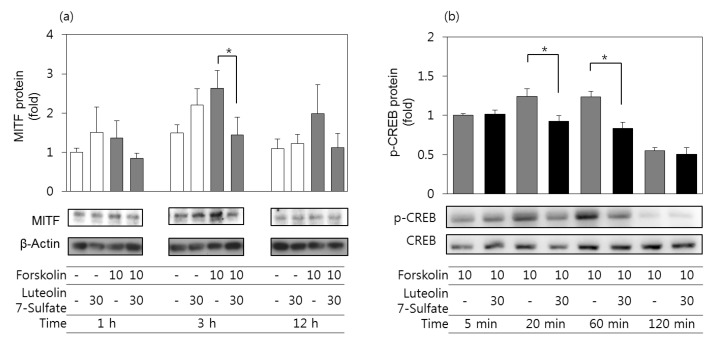
Effects of luteolin 7-sulfate on the protein levels of microphthalmia-associated transcription factor (MITF) and the phosphorylation of cAMP-responsive element binding protein (CREB). B16-F10 cells were stimulated B16-F10 cells were pretreated with 30 μM luteolin 7-sulfate and stimulated with 10 μM forskolin for the indicated time. Western blotting was performed for MITF and β-actin (**a**), and for phospho-CREB (Ser^133^) and total CREB (**b**). The means ± SEs of three independent experiments are presented. * *p* < 0.05.

The protein expression levels of MITF in B16-F10 cells were assessed by western blots. As shown in Figure 4A, the protein expression level of MITF increased after 3 h of treatment with forskolin, and the changes were attenuated in cells pretreated with 30 μM luteolin 7-sulfate. We further assessed the phospho-CREB protein levels in B16-F10 cells stimulated by forskolin in the absence and presence of luteolin 7-sulfate. As shown in Figure 4B, phospho-CREB (Ser133) increased 20 min after forskolin stimulation and remained elevated by 60 min, as determined by western blots, whereas no significant changes were observed for total CREB protein levels. The forskolin-stimulated phosphorylation of CREB was attenuated in cells pretreated with 30 μM luteolin 7-sulfate. 

### 3.6. Effects of Luteolin, Luteolin 7-Sulfate, and Arbutin on the Viability and the Melanin Contents of HEMs

**Figure 5:**
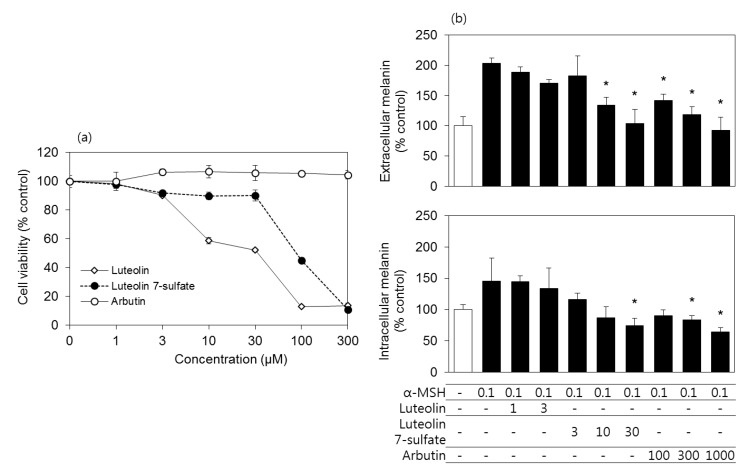
Effects of luteolin, luteolin 7-sulfate, and arbutin on the viability and melanin contents of human epidermal melanocytes. (**a**) Cells were treated with luteolin, luteolin 7-sulfate, or arbutin at varied concentrations, and the cell viability was determined after 48 h. (**b**) Cells were treated with luteolin, luteolin 7-sulfate, or arbutin at their non-toxic concentration ranges for 60 min and stimulated with 0.1 μM α-melanocyte-stimulating hormone (α-MSH), followed by a 48 h incubation. This procedure was repeated three times with medium change for 6 d. Extra- and intracellular melanin contents were corrected for total protein content of the cells. The means ± SEs of three independent experiments are presented. * *p* < 0.05 vs. α-MSH alone.

To verify the findings from B16-F10 cell experiments, assays for cell viability and melanin contents were repeated using primary HEMs. As shown in Figure 5A, the cytotoxicity of luteolin 7-sulfate was much weaker than luteolin in HEMs. As shown in Figure 5B, an α-MSH treatment for 6 d increased both the extra- and intracellular melanin contents of HEMs, and these increases were reduced by luteolin 7-sulfate at 10 times lower concentrations than arbutin.

## 4. Discussion

Luteolin, a kind of flavonoid, has various pharmacological activities, including anti-inflammatory, antimicrobial, and anticancer activities [30]. It inhibits melanin synthesis through the inhibition of TYR catalytic activity [31] and TYR expression, mediated by cAMP-dependent pathways [32]. Luteolin 7-sulfate is an uncommon form of flavonoid found only in a few species of plants, such as *Phyllospadix iwatensis* Makino and *Zostera marina* L. [33,34]. Previously, luteolin 7-sulfate was shown to have a higher TYR inhibiting activity and lower cytotoxicity than luteolin [19]. Using the synthetic compound, the current study additionally showed that luteolin 7-sulfate attenuated the expression of TYR at the mRNA and protein levels. The dual mechanism of luteolin 7-sulfate attenuating both the synthesis of new TYR proteins and the catalytic activity of pre-existing TYR proteins would be advantageous properties of luteolin 7-sulfate as a melanogenesis inhibitor. In addition, luteolin 7-sulfate was less cytotoxic than luteolin, and it was more potent in the inhibition of cellular melanin synthesis compared with arbutin, a well-known melanogenesis inhibitor [29].

α-MSH is a peptide hormone and its stimulatory role in melanogenesis is well established [3,35,36]. α-MSH acts as an agonist of the melanocortin 1 receptor and its binding to the receptor leads to the activation of adenylate cyclase, resulting in cAMP production. Then, protein kinase A phosphorylates CREB, involved in the activation of MITF, which directs melanogenesis by promoting the gene expression of TYR and other melanogenic enzymes. Forskolin directly activates adenylate cyclase, leading to MITF activation and TYR expression through a similar signaling pathway. The current study showed that luteolin 7-sulfate attenuated both the activity and protein level of TYR in cells stimulated by either α-MSH or forskolin. In addition, luteolin 7-sulfate attenuated the mRNA and protein expressions of MITF and TYR stimulated by forskolin. Finally, luteolin 7-sulfate attenuated the phosphorylation of CREB stimulated by forskolin. Therefore, the antimelanogenic effects of luteolin 7-sulfate could be attributed at least partly to the intervention of a CREB- and MITF-mediated signaling pathway, leading to TYR gene expression. 

B16-F10 cells are derived from murine melanoma, and widely used for research as a model of skin pigmentation as well as skin cancer [16,17,18]. B16-F10 cells have all the elements necessary for melanin synthesis in response to the melanogenic signals, and have a higher melanin production than the amelanotic human melanoma cell line A375 cells [37]. In addition, B16-F10 cells grow well in general culture media and are relatively easier to cultivate than HEMs, which require very expensive specialized media for growth. Thus, B16-F10 cells are considered to be a good substitute or complement for HEMs, especially for melanogenesis research. B16-F10 cells were used for most of the experiments in this study, and some critical experiments (cell viability and melanin content analysis) were reproduced in HEMs. However, it is preferable that all other experiments should be reaffirmed in HEMs, and future studies are needed to verify the critical results of this study in three-dimensional cultured skin tissue and in vivo experiments.

Various flavonoids show different effects on melanogenesis [38]. Luteolin inhibited melanin synthesis activity [31,32], but apigenin stimulated melanogenesis [39]. Quercetin showed opposite effects on melanin synthesis in different experiments [40,41]. Some flavonoids, such as quercetin, inhibited cAMP phosphodiesterase [42], and others, such as luteolin, inhibited adenylate cyclase [32]. Thus, it is plausible that each flavonoid could regulate melanogenesis differently by increasing or decreasing cAMP concentrations in cells. Further studies are needed to examine the effects of luteolin 7-sulfate on the activities of adenylate cyclase and cAMP phosphodiesterase. 

The cell uptake of sulfated flavonoids was lower than free flavonoids, but comparable to that of flavonoid glycosides [43]. It is known that flavonoids are rapidly conjugated with sulfate, and sulfated flavonoids can be converted to free flavonoids by sulfatase [44]. Therefore, it is not clear which of luteolin or luteolin 7-sulfate is the active form acting on the targets in cells. Nonetheless, the present study showed that more safe and effective inhibition of cellular melanogenesis could be achieved by treating cells with luteolin 7-sulfate rather than luteolin.

## 5. Conclusions

In conclusion, using the synthetic luteolin 7-sulfate the present study verified its antimelanogenic effects originally found using luteolin 7-sulfate isolated from *Phyllospadix iwatensis*. It also suggests a new action mechanism of luteolin 7-sulfate suppressing the expression of TYR involved in melanogenesis. This study showed that small modification of flavonoids, i.e., luteolin to luteolin 7-sulfate, could significantly alter safety in melanocytic cells. Luteolin 7-sulfate rather than luteolin is considered to be a promising candidate for antimelanogenic agents for the control of abnormal skin pigmentation. 

## Figures and Tables

**Table 1:** Nuclear magnetic resonance (NMR) data of luteolin 7-sulfate and luteolin ^1^.

No.	Luteolin 7-Sulfate	Luteolin	Luteolin 7-Sulfate	Luteolin
	δ_H_ Multiplicity (J = Hz)		δ_C_	
2			164.3	163.9
3	6.76 s	6.67 s	103.0	102.9
4			182.0	181.7
5			160.5	161.5
6	6.52 d (2.1)	6.19 d (2.1)	102.0	98.9
7			159.5	164.2
8	7.03 d (2.1)	6.45 d (2.1)	97.5	93.9
9			156.3	157.3
10			105.6	103.7
1’			121.3	121.6
2’	7.47 d (2.1)	7.42 ^2^	113.3	113.4
3’			145.7	145.8
4’			149.8	149.7
5’	6.89 d (8.4)	6.90 d (8.4)	116.1	116.1
6’	7.46 dd (8.4, 2.1)	7.42 ^2^	119.1	119.0
5-OH	12.87 s	12.98 s		

^1^ Measured at 700 and 175 MHz; obtained in dimethyl sulfoxide (DMSO)-d_6_ with tetramethylsilane (TMS) as an internal standard. ^2^ Overlapping signals.
